# The New Orleans Dental College

**Published:** 1871-03

**Authors:** 


*The New Orleans Dental College.*—The commencement exercises of the New Orleans Dental College, were held on the evening of the 7th of March, at the rooms of the College on Carondelet street. The lecture room of the institution was filled by a company of intelligent ladies and gentleman, who witnessed the exercises with great interest. Among those assembled were a number of members of the dental profession in New Orleans.

The exercises were opened with prayer by Rev. C. S. Hilton, of Christ Church. Dr. J. S. Harrison, one of the Faculty of the College, then read an interesting lecture, the subject being "Incentives to the study of the Natural Sciences." The lecture evidenced great research, and the various subjects it embraced were ably treated. It is to be regretted that the Profeseor omitted some portions of the lecture from the unfounded fear of taking up too much time. Dr. Harrison at the close of the lecture, made a few judicious remarks appropriate to the occasion, and addressed more particularly to the students of the college.

The Dean of the Faculty, Dr. J. S. Knapp, then made a short address to the graduating class, and conferred the degree of Doctor of Dental Surgery on five young gentleman: John A. Arrington, of Tennessee; Thomas D. French, of Mississippi; Herbert Norman, of South Australia; Sidney M. Cook, of Louisiana; and Charles P. Angell, of Louisiana. Honorary degrees were conferred on Messrs. S. J. Cobb, of Tennessee; Rufe Waldo Thornton, of Georgia; and Joseph C. Turner, of Alabama.

An address on behalf of the students was then delivered by Mr. Herbert Norman, of South Australia, one of the graduating class. The address was listened to with attention, and received with plaudits.

Not the least interesting feature of the occasion was the bestowal of splendid boquets upon the graduates by young ladies in the assembly. A few remarks from Dr. Harrison terminated the exercises.

